# Investigating the Use of Ultraviolet Light Emitting Diodes (UV-LEDs) for the Inactivation of Bacteria in Powdered Food Ingredients

**DOI:** 10.3390/foods10040797

**Published:** 2021-04-08

**Authors:** Laura Nyhan, Milosz Przyjalgowski, Liam Lewis, Máire Begley, Michael Callanan

**Affiliations:** 1 Department of Biological Sciences, Munster Technological University, T12 P928 Cork, Ireland; l.nyhan@mycit.ie (L.N.); maire.begley@cit.ie (M.B.); 2 Centre for Advanced Photonics and Process Analysis, Munster Technological University, T12 P928 Cork, Ireland; milosz.przyjalgowski@cit.ie (M.P.); liam.lewis@cit.ie (L.L.)

**Keywords:** ultraviolet, LED, inactivation, bacteria, powder, foods

## Abstract

The addition of contaminated powdered spices and seasonings to finished products which do not undergo further processing represents a significant concern for food manufacturers. To reduce the incidence of bacterial contamination, seasoning ingredients should be subjected to a decontamination process. Ultraviolet light emitting diodes (UV-LEDs) have been suggested as an alternative to UV lamps for reducing the microbial load of foods, due to their increasing efficiency, robustness and decreasing cost. In this study, we investigated the efficacy of UV-LED devices for the inactivation of four bacteria (*Listeria monocytogenes, Escherichia coli, Bacillus subtilis and Salmonella* Typhimurium) on a plastic surface and in four powdered seasoning ingredients (onion powder, garlic powder, cheese and onion powder and chilli powder). Surface inactivation experiments with UV mercury lamps, UVC-LEDs and UVA-LEDs emitting at wavelengths of 254 nm, 270 nm and 365 nm, respectively, revealed that treatment with UVC-LEDs were comparable to, or better than those observed using the mercury lamp. Bacterial reductions in the seasoning powders with UVC-LEDs were less than in the surface inactivation experiments, but significant reductions of 0.75–3 log_10_ colony forming units (CFU) were obtained following longer (40 s) UVC-LED exposure times. Inactivation kinetics were generally nonlinear, and a comparison of the predictive models highlighted that microbial inactivation was dependent on the combination of powder and microorganism. This study is the first to report on the efficacy of UV-LEDs for the inactivation of several different bacterial species in a variety of powdered ingredients, highlighting the potential of the technology as an alternative to the traditional UV lamps used in the food industry.

## 1. Introduction

The microbial contamination of powdered ingredients is not considered a major problem due to the limitation of growth by the low water activity (a_w_) value. However, the addition of contaminated raw powder material to ready-to-eat (RTE) foods may result in the contaminants multiplying to high levels, thus posing a risk to public health. Van Doren et al. (2013) undertook a review of the Centers for Disease Control and Prevention’s Foodborne Disease Outbreak Surveillance (CDCs FDOSS) System, finding that between 1973 and 2010, 14 reported foodborne outbreaks that were attributed to the consumption of contaminated spices such as red pepper and curry powder occurred across 10 countries and resulted in 1946 illnesses, 128 hospitalisations and 2 deaths [1]. *Salmonella enterica* and *Bacillus cereus* were identified as the main causative agents. Seventy per cent of illnesses were attributed to consumption of RTE foods prepared with spices which were applied after the food manufacturing pathogen reduction step, while in 75% of outbreaks, it was reported that no pathogen reduction step had been applied to the spice. Furthermore, the authors identified an additional seven spice-related foodborne outbreaks which lacked microbiological or epidemiological evidence and therefore did not meet the inclusion criteria of the study, highlighting the probability that the number of foodborne outbreaks associated with consumption of contaminated spices is under-reported [1]. Along with this, studies have found dried herbs and spices to be contaminated with various microorganisms including *Salmonella* spp., *Clostridium perfringens*, *Escherichia coli*, *Staphylococcus aureus* and *Enterobacter* spp. at the point of retail [2,3,4]. Thus, spices and seasonings should be subject to a decontamination process in order to protect the consumer and prevent foodborne disease.

Given the serious consequences of foodborne infection and the economic costs associated with destroying contaminated batches and product recalls, it is not surprising that food manufacturers are seeking novel approaches for the control of foodborne pathogens. Various methods have been used to reduce microbial levels in powdered seasonings including thermal processing and irradiation; however each of these methods has limitations. Studies have shown heat treatment to be less effective for microbial decontamination in low-moisture foods such as powders and spices [5,6], while high temperatures can alter the characteristics of the powders such as the flavour, colour and aroma [7,8]. Although irradiation is an effective method for reducing the microbial load of spices, studies have reported negative impacts on the sensory properties of powders, such as a change in appearance, difference in aroma and off-flavours [9,10]. Therefore, alternative decontamination processes are required, one of which could be UVC-LED radiation.

UV radiation is a well-established method for the reduction or elimination of pathogens from foods [11,12,13,14] and has also been tested for the decontamination of powdered ingredients such as flour powdered infant formula (PIF), black pepper and powdered red pepper [15,16,17,18,19,20,21,22]. The UV spectrum is subdivided into the UVA (315–400 nm), UVB (280–315 nm) and UVC (<280 nm) ranges, each with a specific effect on microorganisms. As maximum DNA absorption of UV light occurs at the peak wavelength of 260–265 nm, UVC radiation is usually the most effective for microbial inactivation [23]. Due to this, low-pressure (LP) or medium-pressure (MP) mercury lamps emitting UVC light at a wavelength of 254 nm have traditionally been used for decontamination; however, these lamps have several disadvantages, including a relatively large footprint and the requirement of a warm-up period prior to use [24]. In addition, they pose a health risk to consumers due to possible breakage of the lamp and lamp sleeve and, consequently, the potential release of mercury particles onto the food products undergoing treatment [25], while there is a risk of mercury waste accumulation if not disposed of correctly, which can have damaging effects on human health and the environment [26].

UV light-emitting diodes (UV-LEDs) have emerged as an alternative to traditional UV lamps in recent years. LEDs are composed of layers of semiconductor material which emit light when an electrical current is applied [27]. UV-LEDs are a more sustainable source of energy than UV lamps as they do not use mercury, they can reach maximum output power instantaneously and are becoming increasingly more efficient and economically viable as time goes on. Moreover, their compactness and robustness have highlighted the potential of UV-LEDs as a cost-effective inactivation technology within the food industry [28]. In recent years, UV-LED radiation has been applied to liquid beverages such as fruit juices [29,30,31] and solid food products such as cheese, lettuce, cabbage, tuna fillets and chicken [24,32,33,34] for the reduction of foodborne pathogens. Despite this, the efficacy of UV-LEDs for the inactivation of bacteria in powdered ingredients remains largely uninvestigated, with just two studies found in the literature which investigated the efficacy of UV-LEDs for the inactivation of bacteria in low a_w_ foods, both of which focused on heat resistant strains of *Salmonella* spp. in wheat flour [35,36]. Therefore, the objective of this study was to firstly compare the performance of a UV lamp to UV-LEDs for the surface inactivation of four bacteria (*Listeria monocytogenes*, *E. coli*, *Bacillus subtilis* and *Salmonella* Typhimurium) and following this, investigate the efficacy of UVC-LEDs for inactivation of the bacterial strains in four different seasoning powders.

## 2. Materials and Methods

### 2.1. UV Devices

**Figure 1:**
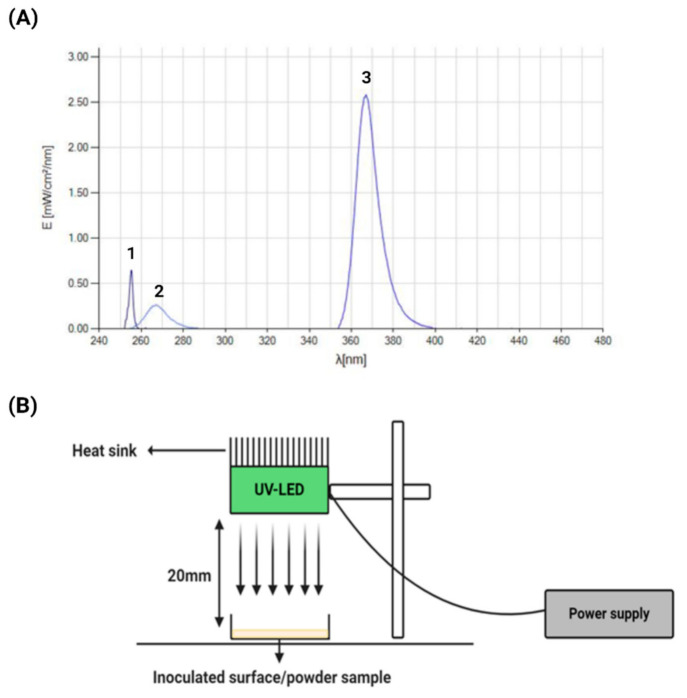
(**A**) Emission profiles of (1) mercury lamp at 254 nm; (2) UVC-LED at 270 nm; (3) UVA-LED at 365 nm. (1) and (2) were measured at 20 mm distance while (3) was measured at 100 mm distance. (**B**) Schematic diagram of the UV-LED experimental set-up. This image was created with BioRender.

The UV and UV-LED devices used in this study were assembled by the Centre for Advanced Photonic and Process Analysis (CAPPA), MTU, Cork. Two types of UV-LED devices were used, emitting at both UVC and UVA wavelengths. The 270 nm UVC LED-based lamps were built around KL265-35R-SM-GD Crystal IS devices (Crystal IS, New York, NY, USA) and the LEDs were driven at a maximum current rating of 300 mA. The 365 nm UVA LED-based lamps were built around LED Engin LZ1 UV 365 nm Gen2 Emitter devices (Osram, Munich, Germany) and driven at a maximum current rating of 1 A. For comparison purposes, a traditional 254 nm mercury lamp was also included in the study, based around 100 mm low pressure Rexim MCCUV-CV-100 × 8 × 100 Hg bulbs (Rexim, Watertown, MA, USA) with 12VDC INV-1L-12V inverters. The emission spectra of each was measured using a UVPad E radiometer (Opstytec Dr. Groebel, Ettlingen, Germany) (Figure 1A). The distance between each of the emitters and the test samples in this study was set at 20 mm for both surface and powder inactivation experiments (Figure 1B).

### 2.2. Bacterial Strains and Growth Conditions

The strains used in this study were obtained from the MTU Cork culture collection. *L. monocytogenes* LO28, *Salmonella enterica* subsp. *enterica* serovar Typhimurium (*S*. Typhimurium) ATCC 49416 and *Bacillus subtilis* subsp. *Spizizenii* (*B. subtilis)* ATCC 6633 were grown aerobically in brain heart infusion (BHI) broth (LabM, Lancashire, UK) at 37 °C. *E. coli* DH5α*lux* contains a plasmid with a kanamycin resistance gene and was grown at 37 °C in Luria-Bertani (LB) broth (LabM, Lancashire, UK) supplemented with 100 µg/mL kanamycin (Merck, Darmstadt, Germany). Stocks of all strains were maintained at −20 °C and −80 °C in a final concentration of 40% glycerol (Merck, Darmstadt, Germany).

### 2.3. Sample Preparation

#### 2.3.1. Petri Dish Surfaces

A 1 mL sample from overnight cultures of each strain was centrifuged at 5000× *g* for 10 min, washed and resuspended in an equal volume of ¼ strength Ringers’ solution (Merck, Darmstadt, Germany). Washed cultures were diluted to a starting concentration of ~10^7^ CFU/mL. Sterile cotton swabs were soaked in the standardised cultures and were used to transfer the inoculum to a small circular area (diameter of 11 mm) in the centre of a sterile Petri dish. Inoculated Petri dishes were left to dry and cultures were re-applied two more consecutive times, resulting in an inoculation level of approx. 10^4^ CFU.

#### 2.3.2. Seasoning Powders

Four seasoning powders (garlic, onion, cheese and onion, and chilli) were sourced from a food manufacturing facility and used in the current study. Particle size of the powders were determined using a Horiba XploRA™ plus confocal Raman microscope. Powders were dispersed over a standard microscope slide. A mosaic image was created with a 50 × objective over a 2 × 2 mm area and processed using the ParticleFinder™ module in the Horiba LabSpec 6 software package. Briefly, this software analyses and counts particles in the image, measuring the particle diameter within defined limits and error margins. A statistical fit is then applied to the size distribution of the particles, which may show single or bimodal distribution. In the current study, approximately 10^2^–10^3^ particles were detected per powder sample. The following imaging processing steps were applied prior to exporting the particle size data: remove edge particle, fill holes, erode filter: 3 × 3 and close filter: 3 × 3. The water activity (a_w_) of the samples was measured at 25 °C using a LabMaster-aw neo water activity meter (Novasina AG, Lachen, Switzerland) (Table 1).

Prior to testing, the absence of the four target microorganisms was confirmed by diluting 1 g of powder in 9 mL of ¼ strength Ringer’s solution and plating onto mannitol yolk polymyxin (MYP) agar (Oxoid), *Listeria* selective agar (LSA) (Merck, Darmstadt, Germany), xylose lysine deoxycholate (XLD) agar (LabM, Lancashire, UK) and eosin methylene blue (EMB) agar (LabM, Lancashire, UK) for selection of *B. subtilis*, *L. monocytogenes*, *S.* Typhimurium and *E. coli*, respectively. Samples were also plated onto BHI agar to assess background microbiota. Powders were inoculated following the method of Callanan et al. (2012) with some modifications [37]. Briefly, overnight cultures (40 mL) of each strain were centrifuged at 10,000× *g* for 10 min, the supernatant was discarded, and excess liquid removed from the pellet using a pipette. Pellets were air dried in a biological safety cabinet for 1.5 h. Powders were inoculated by mixing the dried pellet with 10 g aliquots of the powders using a sterile glass rod until a total of 50 g of powder was inoculated, resulting in an initial population of between 5 and 6 log_10_ CFU/g. To assess even distribution of the inoculum, 5 samples were taken from different areas of each inoculated powder samples and enumerated, with variations (standard deviation) of between 0.3 and 0.5 log_10_ CFU/g observed. Samples were stored in containers aerobically at room temperature for at least 48 h prior to use.

### 2.4. UV and UV-LED Inactivation

All UV inactivation experiments were performed in a Class II biological safety cabinet at room temperature. Samples were placed on a stainless-steel surface at a distance of 20 mm from the UV or UV-LED source. For surface decontamination experiments, the inoculated Petri dish surfaces were exposed to UV (mercury lamp), UVC-LED and UVA-LED sources emitting at 254 nm, 270 nm and 365 nm, respectively, with exposure times of 5 s, 10 s, 20 s and 40 s corresponding to the doses shown in Table 2. For treatment of powders, 1 g of inoculated powder was distributed in a thin layer onto the surface of a styrene container with an area of 36.5 cm^2^ (73 mm × 50 mm × 11 mm), resulting in a powder layer thickness not exceeding 1.5 mm. Powders were exposed to UVC-LED radiation for treatment times of 5–40 s.

### 2.5. Bacterial Enumeration

Following UV and UV-LED exposure, surviving cells were recovered from the inoculated Petri dishes by soaking fresh sterile swabs in 1 mL of ¼ strength Ringers’ solution and swabbing the inoculated area three times. The recovered cells were serially diluted in 900 µL of ¼ strength Ringers’ solution and 100 µL aliquots of dilutions were spread plated onto BHI agar for *B. subtilis*, *L. monocytogenes* and *S.* Typhimurium or LB agar supplemented with 100 µg/mL kanamycin for *E. coli* DH5α*lux*. Following UVC-LED treatment of the powder samples, samples were serially diluted ¼ strength Ringers’ solution. As the powders provided were not sterile and contained low levels of background microbiota (<10^2^ CFU/g), 100 µL aliquots of the sample dilutions were spread plated onto MYP agar, LSA, XLD agar or LB agar supplemented with 100 µg/mL kanamycin for selection of *B. subtilis*, *L. monocytogenes*, *S.* Typhimurium and *E. coli*, respectively. Plates were incubated at 37 °C for 48 h. Following incubation, colonies were counted and results were expressed as the mean log_10_ CFU/mL(g) ± standard deviation.

### 2.6. Modelling of Bacterial Inactivation Kinetics

#### 2.6.1. Log-Linear Model

The log-linear model is based on traditional first-order inactivation kinetics. It assumes that cells have equal susceptibility and that lethality occurs randomly over time during the inactivation treatment. The model equation is written as follows:

(1) $$N_{t}=\;N_{0}\;\exp\left(-k_{maxB}\cdot t\right)$$

where *N_t_* is the population at time *t* (CFU/g), *N*_0_ is the population at time 0, *k_maxB_* is the maximum specific inactivation rate (s^−1^) and *t* is the time (seconds) [38].

#### 2.6.2. Biphasic Model

Described by Cerf (1977), the biphasic model consists of two phases—an initial log-linear decrease (first-order kinetics) due to inactivation of a sensitive microbial population, followed by a second, slower rate of decrease of a more stress-resistant microbial population (tail):

(2) $$log_{10}\;N_{t}=log_{10}\;N_{0}+log_{10}\left(f\cdot \exp\left(-k_{max1}\cdot t\right)+\left(1-f\right)\cdot \exp\left(-kmax_{2}\cdot t\right)\right)$$

where *N_t_*, *N*_0_ and *t* are as defined above, *f* and (1 − *f*) are the UV-resistant and UV-sensitive population fractions, respectively. *K_max_*_1_ and *K_max_*_2_ (s^−1^) are the maximum specific inactivation rates of the UV sensitive and the UV resistant populations, respectively [39].

#### 2.6.3. Weibull Model

Due to heterogeneity in a sample, bacterial strains may not always follow first-order kinetics. Following UV light treatment, inactivation curves generally exhibit a sigmoidal shape and may display concavity or convexity behaviours, which can be described by the Weibull model:

(3) $$Log_{10}\left(\frac{\;N_{t}}{\;N_{0}}\right)\;=\;-{\left(\frac{t}{\delta }\right)}^{p}$$

where *Nt*, *N*_0_ and *t* are as defined above, *δ* is the scale parameter and *p* is the shape parameter. *p* < 1 represents upward concavity of the curve, indicating stress adaptation of surviving microorganisms following UV treatment. *p* > 1 represents downward concavity, indicating that the cells become increasingly damaged with increasing treatment time. *p* = 1 represents a linear curve [40].

#### 2.6.4. Geeraerd shoulder–tail Model

Similar to the log-linear model, the Geeraerd model is based on first-order inactivation kinetics but includes additional parameters for shoulders and tailing. The model is described as follows:

(4) $$N_{t}=\left(N_{0}-N_{res}\right)\cdot \exp\left(-k_{max}\cdot t\right)\;\frac{\exp\left(-k_{max}\cdot SL\right)}{1+\exp\left(\left(-k_{max}\cdot SL\right)-1\right)\exp\left(-k_{max}\cdot t\right)}+N_{res}$$

where *Nt*, *N*_0_ and *t* are as defined above, *N_res_* is the UV resistant population, *k*_max_ is the maximum specific inactivation rate (s^−1^) and SL is the parameter representing the shoulder length (seconds). If log-linear kinetics with and without shoulder/tailing are observed, SL or N_res_ can be equal to zero, resulting in reduced forms of the model, namely log-linear + shoulder and log-linear + tail [41,42].

Data fitting was performed using the GInaFiT application version 1.7 [42] in Microsoft Excel. Goodness of fit was determined using the root mean square error (RMSE) and the adjusted coefficient of determination (R^2^_adj_).

### 2.7. Statistical Analysis

All experiments were carried out using three biological replicates and data are expressed as the mean ± standard deviation (SD). CFU data were transformed to log_10_ prior to analysis. Statistical analysis was performed using R Studio software. Data were analysed using one-way analysis of variance (ANOVA) with post hoc comparison using Tukey’s multiple comparisons test. Asterisks rating of *, ** or *** indicates statistically significant differences between groups (*p* ≤ 0.05, *p* ≤ 0.005 or *p* ≤ 0.001, respectively).

## 3. Results

### 3.1. UV-LED Surface Inactivation of Microorganisms

**Figure 2:**
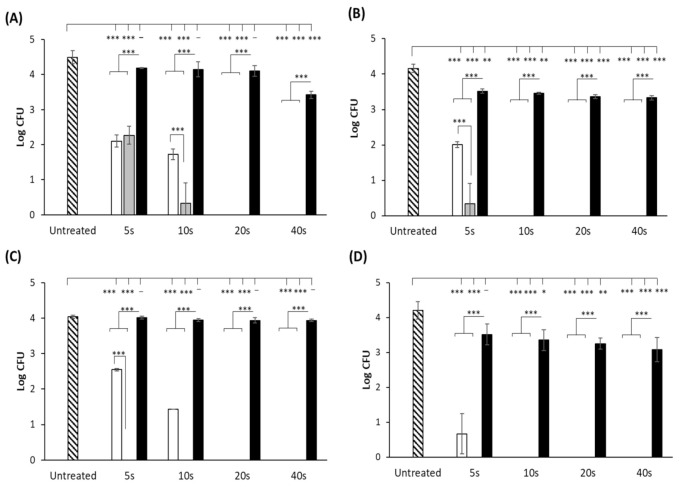
Mean log CFU values of (**A**) *L. monocytogenes;* (**B**) *E. coli;* (**C**) *B. subtilis;* (**D**) *S.* Typhimurium following exposure to UV light at wavelengths of 254 nm (white bars) and UV-LED light at 270 nm (grey bars) and 365 nm (black bars) on a plastic Petri dish surface. Error bars represent the standard deviation of replicate experiments. Absence of a bar indicates that bacteria were reduced to below detection level (10 CFU). *** denotes *p* ≤ 0.001, ** denotes *p* ≤ 0.005, * denotes *p* ≤ 0.05 and – denotes no statistical significance

Log reductions of each bacterial strain (log CFU) on a plastic surface following exposure to UV, UVC-LED and UVA-LED sources at wavelengths of 254 nm, 270 nm and 365 nm, respectively, are shown in Figure 2. The results showed that the cell numbers of all four strains were significantly reduced following 5 s exposure to the traditional 254 nm mercury lamp (*p* < 0.05), with *E. coli, B. subtilis* and *S.* Typhimurium reduced below the limit of detection (10 CFU) after 10 s exposure, and *L. monocytogenes* after 20 s exposure. Similar to the mercury lamp, a 5 s exposure to the UVC-LED also resulted in a significant decrease in cell numbers of all strains (*p* < 0.05). Reductions of 2.2 and 3.8 log_10_ CFU were observed for *L. monocytogenes* and *E. coli, respectively*, while *B. subtilis* (Figure 2C) and *S.* Typhimurium (Figure 2D) proved the most susceptible to UVC-LED inactivation, with cell numbers reduced to below the detection limit, a reduction of over 4 log_10_ CFU for each strain. A 10 s UV-LED exposure was sufficient for complete inactivation of *E. coli* cells, while *L. monocytogenes* again proved to be the most tolerant of the four strains, requiring a longer exposure time of 20 s for inactivation. In most cases, treatment of the bacteria with the UVC-LEDs resulted in significantly higher log reductions than those obtained following treatment with the mercury lamp. The only exception to this was the 5 s treatment of *L. monocytogenes*, whereby the cell numbers were reduced to similar levels (*p* > 0.05) following exposure to both the mercury and the UVC-LED lamps (Figure 2A). Of the three lamps tested, the 365 nm UVA-LED was the least effective for bacterial inactivation. Following 40 s exposure, reductions of approx. 1.07, 0.83 and 1.12 log_10_ CFU were observed for *L. monocytogenes*, *E. coli* and *S.* Typhimurium, respectively, while the lamp had little to no effect on *B. subtilis*, with ≤0.1 log_10_ reduction observed.

### 3.2. UVC-LED Inactivation of Microorganisms in Powdered Ingredients

**Figure 3:**
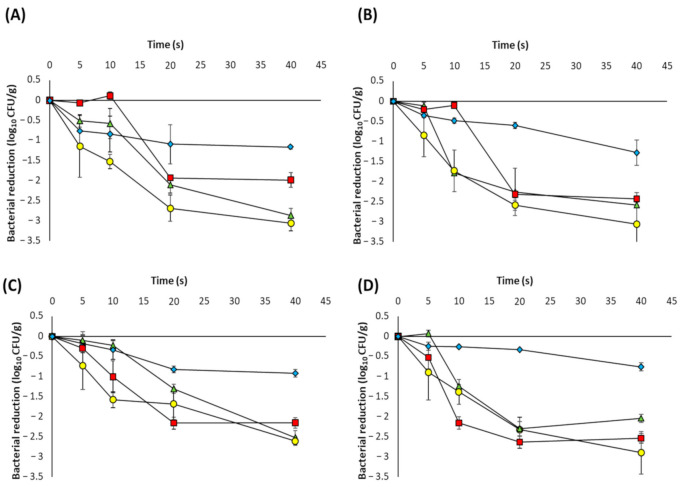
Inactivation curves of (**A**) *L. monocytogenes*; (**B**) *E. coli*; (**C**) *B. subtilis;* (**D**) *S*. Typhimurium following exposure to UVC-LED light at 270 nm in garlic powder (▲ green triangle), onion powder (█ red square), cheese and onion powder (● yellow circle) and chilli powder (♦ blue diamond). Data are represented as the mean ± standard deviation of three biological replicates.

The surface inactivation experiments demonstrated that the performance of the 270 nm UVC-LED lamp was significantly superior to that of the 365 nm UVA-LED lamp, and resulted in bacterial reductions which were comparable to, or in most cases, better than those obtained using the mercury lamp. Therefore, only the UVC-LEDs were utilised in the powdered ingredient inactivation experiments. The inactivation curves of the four bacteria in each of the four seasoning powders are shown in Figure 3. The results showed powder type has an impact on bacterial inactivation. In particular, the cells were more susceptible to UVC-LED light in the cheese and onion powder rather than the onion or garlic powders. This is particularly evident in the case of *L. monocytogenes*, where a 20 s exposure time was required to reduce recoverable cell numbers in onion powder and garlic powder to the same levels obtained with a 10 s exposure in cheese and onion powder (Figure 3A). In chilli powder, despite a significant reduction in cell numbers for all four strains following 40 s treatment (between 0.75–1.3 log_10_ CFU/g), the overall levels of inactivation were lower than those observed in the other three powders. The physiochemical properties of the powder have been shown to influence UV inactivation efficacy in food matrices [11,33,43]. Therefore, particle size and water activity were determined (Table 1); however, in our study, smaller particle size and higher water activity were not associated with increased inactivation.

### 3.3. Model Fitting

Mathematical models are used to quantify and compare the microbial inactivation efficacy of different technologies. Matrix effects such as shadowing and the shallow penetration of UV radiation means there is no consensus model for UV microbial inactivation kinetics, so we tested the ability of a number of commonly applied mathematical models (log linear, biphasic, Weibull and Geeraerd shoulder–tail) to accurately describe the observed inactivation kinetics. The UVC-LED inactivation profiles (Figure 3) differed depending on the powder and microbe, with some showing a shoulder phase, some a tailing phase and some with both shoulders and tails. In both onion and garlic powders, little or no decrease in cell numbers was observed within the first 5–10 s of treatment (shoulder), followed by a significant reduction after 20 s of UVC-LED treatment (between 1.3–2.6 log_10_ CFU/g), and a final gradual decrease of approx. 0.5–1 log_10_ CFU/g (tail). The data were fitted with the four different inactivation models and the goodness of fit of each model was assessed using the R^2^_adj_ and RMSE values (Table 3). Unsurprisingly, the log-linear model was a poor fit for the data in most of the conditions with the exception of *S*. Typhimurium in chilli powder (R^2^_adj_ = 0.884, RMSE = 0.088).

**Figure 4:**
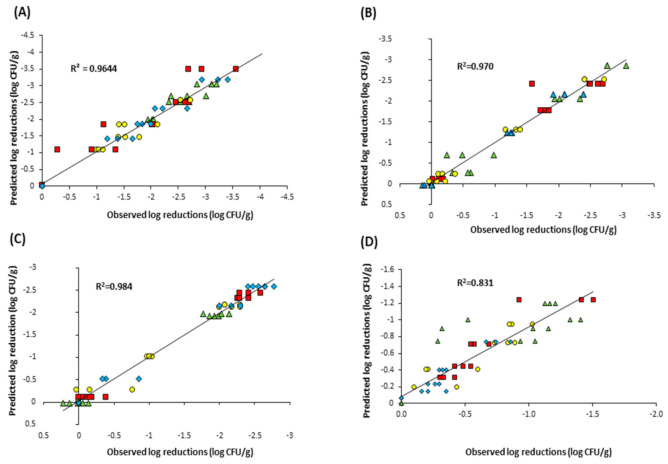
Correlation between experimentally observed log reductions and predicted log reductions of *L. monocytogenes* (▲ green triangle), *E. coli* (█ red square), *B. subtilis* (● yellow circle) and *S. Typhimurium* (♦ blue diamond) as predicted by (**A**) the biphasic model, in cheese and onion powder; (**B**) the Geeraerd shoulder–tail model, in garlic powder; (**C**) the Geeraerd-tail model, in onion powder and (**D**) the biphasic model, in chilli powder.

The biphasic model assumes the presence of two populations in the matrix, a major population which is more susceptible (k_max1_) and a minor population which is more resistant (k_max2_). Therefore, curves which show a sharp initial decline followed by a tailing phase are generally described well by the biphasic model and it was statistically the best fit for *L. monocytogenes* (R^2^_adj_ = 0.975, RMSE = 0.180) and *B. subtilis* (R^2^_adj_ = 0.942, RMSE = 0.217) in cheese and onion powder. In contrast, the behaviour of *E. coli* and *S.* Typhimurium in this powder were best described using the Weibull model which has been used in other studies to describe UV-C inactivation [43,44]. The Geeraerd-shoulder-tail model, which describes inactivation curves incorporating both shoulder and tailing phases, was the statistical best fit for all of the strain data in both garlic powder and onion powder. Interestingly, while this model showed the best goodness-of-fit for the both the garlic and onion powder data, the model could not be fitted to any of the data obtained in the cheese & onion powder, or to the *L. monocytogenes* and *E. coli* data in chilli powder. The correlation between experimentally observed bacterial log reductions and those predicted by the best-fitting model for each of the four powders is illustrated in Figure 4 and suggests that overall, the Geeraerd-shoulder-tail model was the most accurate.

## 4. Discussion

The objective of this study was to investigate the use of UV-LED devices for inactivation of pathogenic microbes in powdered seasoning ingredients. Initially, the efficacy of both UVC-LED (270 nm) and UVA-LED (365 nm) devices for bacterial inactivation was investigated on a Petri dish surface, while a traditional mercury lamp was included for comparison purposes. The results showed that the UVC-LED was at least as effective or in some cases, more effective than the mercury lamp. Kim et al. (2016) reported similar findings, observing significantly higher reductions of *E. coli* O157:H7 in microbiological media with UV-LEDs emitting at 266 nm compared to a mercury lamp [24], while other authors have found similar success with UV-LEDs at wavelengths of 260–285 nm [29,45,46,47,48,49]. This highlights the advantage of using UV-LEDs which can be designed to produce specific wavelengths instead of mercury lamps which can only emit at a single wavelength of 254 nm and have a large environmental footprint.

Santos et al. (2014) investigated the effect of different wavelengths (UVA, UVB and UVC) on the inactivation of nine different bacterial isolates in media, reporting that UVC light was most effective for bacterial inactivation, with the highest survival rates observed following UVA exposure [50]. Likewise, other researchers have made similar observations regarding the inefficacy of UVA light for bacterial inactivation compared to UVC radiation [49,51]. Although some studies have reported significant bacterial reductions in liquids and on food surfaces following UVA-LED treatment [32,35,36,52,53], long exposure times (30–180 min) were required, highlighting the time impracticalities of UVA-LED inactivation. However, as the fluence emitted from the UVA-LED used in the current study was significantly higher than that of the mercury lamp or the UVC-LED source, it was included to investigate whether these increased energy levels would result in a significant reduction in bacterial cell numbers within a short treatment period. Similar to the results observed Hinds et al. (2019), the UVA-LED did not significantly reduce cell numbers of *B. subtilis* [49]; however, significant reductions (approximately 1-log_10_ CFU) in cell numbers of *L. monocytogenes*, *E. coli* and *S*. Typhimurium were obtained after 40 s treatment. Nevertheless, despite the increased energy doses, the performance of the UVA-LED source for bacterial inactivation was inferior to that of the mercury lamp or the UVC-LED, findings which are in agreement with the literature [49,50].

The Gram-positive pathogen *L. monocytogenes* appeared to be more difficult to eradicate on the plastic surface by UVC-LED radiation, requiring 64 mJ/cm^2^ of UVC exposure for complete inactivation, compared to the Gram-negative *E. coli* and *S.* Typhimurium strains which required just 32 mJ/cm^2^ and 16 mJ/cm^2^, respectively. It has been reported that Gram-positive microorganisms tend to be more UV-resistant, requiring higher UV dosages than Gram-negative bacteria for inactivation. Gabriel et al. (2009) found that *L. monocytogenes* was significantly more resistant to UV treatment than *E. coli* and *S.* Enteritidis, with similar observations made by Shin et al. (2016) [47,54]. According to Gayán et al. (2014), differences between the UV-resistance of species can be attributed to several factors including cell wall thickness, cell size and DNA repair efficiency [55].

Bacterial strains were most and least susceptible to UVC-LED inactivation in cheese and onion powder and chilli powder, respectively. Other studies have also shown the efficacy of UV treatment to be dependent on the matrix [11,12,33,43,56], with differences commonly attributed to the ingredient composition and the chemical and physical properties of the food. The particle size of the food sample is one such characteristic which can impact UV-LED inactivation of microorganism. UV radiation penetration of powders is difficult due to the shadowing effect of food particles, which protects the bacterial cells from complete exposure, while bacterial cells can also protect each other from UV light [55], a phenomenon which has been observed in several studies [17,19,20,21]. As the particle sizes of the powders used in this study are generally larger than the typical cell size of each of the target microorganisms it is plausible that the crevices and cavities on the surface of the powders shielded a subpopulation of the microbial cells from the UVC light. Furthermore, the chilli powder used in the present study contained large spice particles which measured approx. 12-fold, 2.3-fold and 2-fold larger than the garlic, onion and cheese and onion particles, respectively; thus, it is likely that those particles physically shielded both the finer spice particles and the bacterial cells from the UVC-LED light, resulting in the lowest bacterial inactivation rates of all powders tested. Condón-Abanto et al. (2016) addressed the problem of shielding in flour by using sample sizes of 1 g and 0.1 g, resulting in reductions of approx. 2-log_10_ and 2.5-log_10_ CFU/g after 60 s treatment, respectively, most likely due to the reduction in the number of particles capable of producing a shadow effect [17]. Ha and Kang (2013, 2014) used a rotational mixer during UV radiation of *E. coli* and *S.* Typhimurium in red pepper powder [19] and *C. sakazakii* in PIF [20] with the aim of increasing the contact surface area of the UV light on the particles; however, the reduction in cell numbers obtained was more than 50 times less than those observed by Condón-Abanto et al. (2016) and in the current study, probably due to the large sample size of 250 g. In our study, we did not observe any significant differences in the log reductions obtained in samples which were stirred at intervals during UV-LED treatment (data not shown).

The fitting of inactivation models to the data confirmed that the bacteria did not show linear behaviour during inactivation, with both shoulders and/or tails observed in most curves and the Geeraerd shoulder–tail model being the most accurate of the models assessed. A number of studies have observed inactivation curves with shoulders following UV treatment of microbes in culture media [17,57,58] and liquid foods such as fruit and vegetable juices [43,59,60,61] However, as each study differs in the choice of UV device and treatment protocol, it is possible that the presence/absence of a shoulder may be dependent on the UV-LED dosage. For example, some studies report bacterial log reductions following a single UV treatment [16,22,56] while Liu et al. (2012) and Arroyo et al. (2017) did not observe shoulders following the inactivation of *C. sakazakii* in PIF; however, higher initial doses were used [15,21]. Similarly, Condón-Abanto et al. (2016) reported inactivation levels of >1.5 log_10_ CFU/g of *S. Typhimurium* in flour during the first 60 s of UVC treatment [17]. Thus, it is possible that the absence of a shoulder in these studies may be due to the high initial UV dosage, leading to significant reductions in cell numbers during the early stages of the inactivation treatment. The tailing phenomenon observed in the inactivation curves can be attributed to resistant subpopulations but it is more likely due to subpopulations that are inaccessible or do not receive the same lethal dose [44] This is due to the previously described shadowing effect of the powder particles preventing complete UV penetration and, subsequently, the survival of a small population of cells. A similar scenario was observed by Gabriel et al. (2020), whereby total log reductions of *E. coli* and *S. aureus* on dried black peppercorns ranged from 1.92–3.60 log_10_ CFU/g after 90 min of UVC treatment, with the majority of inactivation occurring within the first 20 min (0–500 mJ/cm^2^) [62].

As with any food processing treatment, UV radiation can negatively impact the nutritional and organoleptic characteristics of food products. In particular, components which are capable of absorbing UV light, such as vitamin A, vitamin C, riboflavin and food colourings, are the most affected by photochemical reactions [63,64]. UV treatment has also been shown to induce increased lipid oxidation in foods [65,66]. However, few published studies are available which investigate effect of UV-LEDs on food quality characteristics, with most of the research focusing on microbial inactivation. Subedi et al. (2020) observed changes in the gluten structure and oxidation of gluten proteins and in wheat flour following UV-LED treatment at wavelengths ranging from 275–455 nm, with similar results reported by Du et al. (2020) [35,36]. Both Akgün and Unluturk (2017) and Ghate et al. (2016) noted colour changes in fruit juice following UV-LED treatment [29,67], while in contrast, Kim et al. (2017) reported no adverse effects on the nutritional or chemical properties of fresh-cut papaya or mango following UVA-LED exposure [68,69]. As the focus of this study was to investigate the efficacy of UV-LEDs for the microbial decontamination of powders, the impact of the treatment on the powders was not investigated and in general, studies in the literature describing the effect of UV-LED exposure on the nutritional and organoleptic properties of foods are scarce. Thus, it is evident that further work is required to address this area of UV-LED research.

## 5. Conclusions

In conclusion, we have demonstrated the potential of UVC-LEDs as an alternative to traditional UV lamps for bacterial inactivation. UVC-LED emission at 270 nm was just as effective or in some cases more effective than the 254 nm mercury lamp for surface decontamination of four bacterial strains. There are limited data available regarding the use of UV-LEDs and powdered foods and although previous studies have investigated the efficacy of UV-LEDs for inactivation of *Salmonella* spp. in wheat flour, to our knowledge the current study is the first to report the effect of UV-LEDs on four different bacteria in a range of different powdered ingredients. The results showed that bacterial numbers in each of the four powders were reduced significantly in just 40 s, highlighting the power and efficacy of UV-LEDs lamps in comparison to their mercury counterparts.

## Figures and Tables

**Table 1:** Seasoning powder properties.

Powder	Particle Size (µm)	a_w_
Garlic	4.64 ± 2.58	0.358
Onion	24.14 ± 14.57	0.336
Cheese and onion	31.24 ± 14.69	0.371
Chilli (large particles) ^1^	55.75 ± 55.28	0.430
Chilli (fine particles)	15.20 ± 20.99	0.430

^1^ Two particle sizes were measured for chilli powder due to the presence of large and fine particles in the sample.

**Table 2:** Light intensity data for UV mercury lamp (254 nm), UVC-LED (270 nm) and UVA-LED (365 nm) measured at 20 mm at maximum current using a UVPad E radiometer.

Time (s)	UV Radiation Dose (mJ/cm^2^)		
	254 nm	270 nm	365 nm
5	20	16	1700
10	40	32	3400
20	80	64	6800
40	160	128	12,600

**Table 3:** Goodness of fit parameters of the log-linear, biphasic, Weibull and Geeraerd shoulder–tail models for inactivation of bacterial strains in garlic, onion, cheese and onion and chilli powders.

Powder	Microorganism	Inactivation Model							
		Log-Linear		Biphasic		Weibull		Geeraerd Shoulder–Tail	
		R^2^_adj_	RMSE	R^2^_adj_	RMSE	R^2^_adj_	RMSE	R^2^_adj_	RMSE
Garlic	*L. monocytogenes*	0.902	0.357	0.928	0.305	0.906	0.349	**0.947** ^1^	**0.261**
	*E. coli*	0.684	0.645	0.857	0.433	0.766	0.555	**0.938**	**0.286**
	*B. subtilis*	0.959	0.205	-	-	0.962	0.197	**0.985**	**0.121**
	*S.* Typhimurium	0.629	0.627	0.880	0.357	0.701	0.563	**0.982**	**0.137**
Onion	*L. monocytogenes*	0.720	0.533	0.756	0.497	0.701	0.550	**0.986**	**0.121**
	*E. coli*	0.761	0.567	0.789	0.531	0.752	0.577	**0.987**	**0.130**
	*B. subtilis*	0.738	0.487	0.908	0.289	0.812	0.413	**0.956**	**0.200**
	*S.* Typhimurium	0.575	0.742	0.929	0.303	0.771	0.545	**0.979**	**0.164**
Cheese and onion	*L. monocytogenes*	0.514	0.787	**0.975**	**0.180**	0.868	0.410	-	-
	*E. coli*	0.834	0.514	0.929	0.336	**0.937**	**0.316**	-	-
	*B. subtilis*	0.789	0.413	**0.942**	**0.217**	0.935	0.229	-	-
	*S.* Typhimurium	0.788	0.510	0.967	0.202	**0.968**	**0.199**	-	-
Chilli	*L. monocytogenes*	0.368	0.413	0.576	0.339	**0.616**	**0.322**	-	-
	*E. coli*	0.876	0.160	**0.879**	**0.158**	0.874	0.161	-	-
	*B. subtilis*	0.746	0.199	**0.851**	**0.152**	0.787	0.182	0.827	0.164
	*S.* Typhimurium	**0.884**	**0.088**	0.863	0.096	0.877	0.091	0.863	0.096

^1^ Values highlighted in bold represent the best fitting model for each bacteria in each powder.

## Data Availability

The data presented in this study are available on request from the corresponding author.

## References

[1] Van Doren J.M., Neil K.P., Parish M., Gieraltowski L., Gould L.H., Gombas K.L. (2013). Foodborne illness outbreaks from microbial contaminants in spices, 1973–2010. Food Microbiol..

[2] Moreira P.L., Lourencao T.B., Pinto J.P.A.N., Rall V.L.M. (2009). Microbiological quality of spices marketed in the city of Botucatu, Sao Paulo, Brazil. J. Food Prot..

[3] Sagoo S.K., Little C.L., Greenwood M., Mithani V., Grant K.A., McLauchlin J., de Pinna E., Threlfall E.J. (2009). Assessment of the microbiological safety of dried spices and herbs from production and retail premises in the United Kingdom. Food Microbiol..

[4] Sospedra I., Soriano J.M., Mañes J. (2010). Assessment of the microbiological safety of dried spices and herbs commercialized in Spain. Plant. Foods Hum. Nutr..

[5] Laroche C., Fine F., Gervais P. (2005). Water activity affects heat resistance of microorganisms in food powders. Int. J. Food Microbiol..

[6] Santillana Farakos S.M., Frank J.F., Schaffner D.W. (2013). Modeling the influence of temperature, water activity and water mobility on the persistence of *Salmonella* in low-moisture foods. Int. J. Food Microbiol..

[7] Rhim J.W., Hong S.I. (2011). Effect of water activity and temperature on the color change of red pepper (*Capsicum annuum* L.) powder. Food Sci. Biotechnol..

[8] Baechler R., Clerc M.-F., Ulrich S., Benet S. (2005). Physical changes in heat-treated whole milk powder. Lait.

[9] Duncan S.E., Moberg K., Amin K.N., Wright M., Newkirk J.J., Ponder M.A., Acuff G.R., Dickson J.S. (2017). Processes to preserve spice and herb quality and sensory integrity during pathogen inactivation. J. Food Sci..

[10] Jung K., Song B.S., Kim M.J., Moon B.G., Go S.M., Kim J.K., Lee Y.J., Park J.H. (2015). Effect of X-ray, gamma ray, and electron beam irradiation on the hygienic and physicochemical qualities of red pepper powder. LWT Food Sci. Technol..

[11] Holck A., Liland K.H., Carlehög M., Heir E. (2018). Reductions of *Listeria monocytogenes* on cold-smoked and raw salmon fillets by UV-C and pulsed UV light. Innov. Food Sci. Emerg. Technol..

[12] Keklik N., Demirci A., Puri V., Heinemann P. (2012). Modeling the inactivation of *Salmonella* Typhimurium, *Listeria monocytogenes* and *Salmonella* Enteritidis on poultry products exposed to pulsed UV light. J. Food Prot..

[13] McLeod A., Hovde Liland K., Haugen J.-E., Sørheim O., Myhrer K.S., Holck A.L. (2018). Chicken fillets subjected to UV-C and pulsed UV light: Reduction of pathogenic and spoilage bacteria, and changes in sensory quality. J. Food Saf..

[14] Unluturk S., Atilgan M.R., Baysal A.H., Unluturk M.S. (2010). Modeling inactivation kinetics of liquid egg white exposed to UV-C irradiation. Int. J. Food Microbiol..

[15] Arroyo C., Dorozko A., Gaston E., O’Sullivan M., Whyte P., Lyng J.G. (2017). Light based technologies for microbial inactivation of liquids, bead surfaces and powdered infant formula. Food Microbiol..

[16] Cheon H.L., Shin J.Y., Park K.H., Chung M.S., Kang D.H. (2015). Inactivation of foodborne pathogens in powdered red pepper (*Capsicum annuum* L.) using combined UV-C irradiation and mild heat treatment. Food Control..

[17] Condón-Abanto S., Condón S., Raso J., Lyng J.G., Álvarez I. (2016). Inactivation of *Salmonella* Typhimurium and *Lactobacillus plantarum* by UV-C light in flour powder. Innov. Food Sci. Emerg. Technol..

[18] Fine F., Gervais P. (2004). Efficiency of pulsed UV light for microbial decontamination of food powders. J. Food Prot..

[19] Ha J.W., Kang D.H. (2013). Simultaneous near-infrared radiant heating and UV radiation for inactivating *Escherichia coli* O157: H7 and *Salmonella enterica* serovar Typhimurium in powdered red pepper (*Capsicum annuum* L.). Appl. Environ. Microbiol..

[20] Ha J.W., Kang D.H. (2014). Synergistic bactericidal effect of simultaneous near-infrared radiant heating and UV radiation against *Cronobacter sakazakii* in powdered infant formula. Appl. Environ. Microbiol..

[21] Liu Q., Lu X., Swanson B.G., Rasco B.A., Kang D.H. (2012). Monitoring ultraviolet (UV) radiation inactivation of *Cronobacter sakazakii* in dry infant formula using fourier transform infrared spectroscopy. J. Food Sci..

[22] Nicorescu I., Nguyen B., Moreau-Ferret M., Agoulon A., Chevalier S., Orange N. (2013). Pulsed light inactivation of *Bacillus subtilis* vegetative cells in suspensions and spices. Food Control..

[23] Dai T., Vrahas M.S., Murray C.K., Hamblin M.R. (2012). Ultraviolet C irradiation: An alternative antimicrobial approach to localized infections?. Expert Rev. Anti. Infect..

[24] Kim S.J., Kim D.K., Kang D.H. (2016). Using UVC light-emitting diodes at wavelengths of 266 to 279 nanometers to inactivate foodborne pathogens and pasteurize sliced cheese. Appl. Environ. Microbiol..

[25] Clarke S. (2006). Ultraviolet Light Disinfection in the Use of Individual Water Purification Devices.

[26] Hamamoto A., Mori M., Takahashi A., Nakano M., Wakikawa N., Akutagawa M., Ikehara T., Nakaya Y., Kinouchi Y. (2007). New water disinfection system using UVA light-emitting diodes. J. Appl. Microbiol..

[27] Hinds L.M., O’Donnell C.P., Akhter M., Tiwari B.K. (2019). Principles and mechanisms of ultraviolet light emitting diode technology for food industry applications. Innov. Food Sci. Emerg. Technol..

[28] D’Souza C., Yuk H.G., Khoo G.H., Zhou W. (2015). Application of light-emitting diodes in food production, postharvest preservation, and microbiological food safety. Compr. Rev. Food Sci. Food Saf..

[29] Akgün M.P., Ünlütürk S. (2017). Effects of ultraviolet light emitting diodes (LEDs) on microbial and enzyme inactivation of apple juice. Int. J. Food Microbiol..

[30] Lian X., Tetsutani K., Katayama M., Nakano M., Mawatari K., Harada N., Hamamoto A., Yamato M., Akutagawa M., Kinouchi Y. (2010). A new colored beverage disinfection system using UV-A light-emitting diodes. Biocontrol Sci..

[31] Xiang Q., Fan L., Zhang R., Ma Y., Liu S., Bai Y. (2020). Effect of UVC light-emitting diodes on apple juice: Inactivation of *Zygosaccharomyces rouxii* and determination of quality. Food Control..

[32] Aihara M., Lian X., Shimohata T., Uebanso T., Mawatari K., Harada Y., Akutagawa M., Kinouchi Y., Takahashi A. (2014). Vegetable surface sterilization system using UVA light-emitting diodes. J. Med. Investig..

[33] Fan L., Liu X., Dong X., Dong S., Xiang Q., Bai Y. (2020). Effects of UVC light-emitting diodes on microbial safety and quality attributes of raw tuna fillets. LWT Food Sci. Technol..

[34] Haughton P.N., Grau E.G., Lyng J., Cronin D., Fanning S., Whyte P. (2012). Susceptibility of *Campylobacter* to high intensity near ultraviolet/visible 395 ± 5 nm light and its effectiveness for the decontamination of raw chicken and contact surfaces. Int. J. Food Microbiol..

[35] Du L., Jaya Prasad A., Gänzle M., Roopesh M.S. (2020). Inactivation of *Salmonella* spp. in wheat flour by 395 nm pulsed light emitting diode (LED) treatment and the related functional and structural changes of gluten. Food Res. Int..

[36] Subedi S., Du L., Prasad A., Yadav B., Roopesh M.S. (2020). Inactivation of *Salmonella* and quality changes in wheat flour after pulsed light-emitting diode (LED) treatments. Food Bioprod. Process..

[37] Callanan M., Paes M., Iversen C., Kleijn R., Bravo Almeida C., Peñaloza W., Johnson N., Vuataz G., Michel M. (2012). Behavior of *Enterobacter pulveris* in amorphous and crystalline powder matrices treated with supercritical carbon dioxide. J. Dairy Sci..

[38] Bigelow W.D., Esty J.R. (1920). The thermal death point in relation to time of typical thermophilic organisms. J. Infect. Dis..

[39] Cerf O. (1977). Tailing of survival curves of bacterial spores. J. Appl. Bacteriol..

[40] Mafart P., Couvert O., Gaillard S., Leguerinel I. (2002). On calculating sterility in thermal preservation methods: Application of the Weibull frequency distribution model. Int. J. Food Microbiol..

[41] Geeraerd A.H., Herremans C.H., Van Impe J.F. (2000). Structural model requirements to describe microbial inactivation during a mild heat treatment. Int. J. Food Microbiol..

[42] Geeraerd A.H., Valdramidis V.P., Van Impe J.F. (2005). GInaFiT, a freeware tool to assess non-log-linear microbial survivor curves. Int. J. Food Microbiol..

[43] Fenoglio D., Ferrario M., García Carrillo M., Schenk M., Guerrero S. (2020). Characterization of microbial inactivation in clear and turbid juices processed by short-wave ultraviolet light. J. Food Process. Preserv..

[44] Mutz Y.S., Rosario D.K.A., Bernardes P.C., Paschoalin V.M.F., Conte-Junior C.A. (2020). Modeling *Salmonella* Typhimurium inactivation in dry-fermented sausages: Previous habituation in the food matrix undermines UV-C decontamination efficacy. Front. Microbiol..

[45] Cheng Y., Chen H., Sánchez Basurto L.A., Protasenko V.V., Bharadwaj S., Islam M., Moraru C.I. (2020). Inactivation of *Listeria* and *E. coli* by Deep-UV LED: Effect of substrate conditions on inactivation kinetics. Sci. Rep..

[46] Sholtes K.A., Lowe K., Walters G.W., Sobsey M.D., Linden K.G., Casanova L.M. (2016). Comparison of ultraviolet light-emitting diodes and low-pressure mercury-arc lamps for disinfection of water. Environ. Technol..

[47] Shin J.Y., Kim S.J., Kim D.K., Kang D.H. (2016). Fundamental characteristics of deep-UV light-emitting diodes and their application to control foodborne pathogens. Appl. Environ. Microbiol..

[48] Kim D.K., Kim S.J., Kang D.H. (2017). Bactericidal effect of 266 to 279 nm wavelength UVC-LEDs for inactivation of Gram positive and Gram negative foodborne pathogenic bacteria and yeasts. Food Res. Int..

[49] Hinds L.M., Charoux C.M.G., Akhter M., O’Donnell C.P., Tiwari B.K. (2019). Effectiveness of a novel UV light emitting diode based technology for the microbial inactivation of *Bacillus subtilis* in model food systems. Food Control..

[50] Santos A.L., Oliveira V., Baptista I., Henriques I., Gomes N.C.M., Almeida A., Correia A., Cunha Â. (2013). Wavelength dependence of biological damage induced by UV radiation on bacteria. Arch. Microbiol..

[51] Wang C., Lu S., Zhang Z. (2019). Inactivation of airborne bacteria using different UV sources: Performance modeling, energy utilization and endotoxin degradation. Sci. Total Environ..

[52] Lui G.Y., Roser D., Corkish R., Ashbolt N.J., Stuetz R. (2016). Point-of-use water disinfection using ultraviolet and visible light-emitting diodes. Sci. Total Environ..

[53] Prasad A., Gänzle M., Roopesh M.S. (2019). Inactivation of *Escherichia coli* and *Salmonella* using 365 and 395 nm high intensity pulsed light emitting diodes. Foods.

[54] Gabriel A.A., Nakano H. (2009). Inactivation of *Salmonella, E. coli* and *Listeria monocytogenes* in phosphate-buffered saline and apple juice by ultraviolet and heat treatments. Food Control..

[55] Gayán E., Condón S., Álvarez I. (2014). Biological aspects in food preservation by ultraviolet light: A review. Food Bioprocess. Technol..

[56] Stoops J., Jansen M., Claes J., Van Campenhout L. (2013). Decontamination of powdery and granular foods using continuous wave UV radiation in a dynamic process. J. Food Eng..

[57] Gayán E., Mañas P., Álvarez I., Condón S. (2013). Mechanism of the synergistic inactivation of *Escherichia coli* by UV-C light at mild temperatures. Appl. Environ. Microbiol..

[58] Lasagabaster A., Martínez de Marañón I. (2013). Impact of process parameters on *Listeria innocua* inactivation kinetics by pulsed light technology. Food Bioprocess. Technol..

[59] Arroyo C., Gayán E., Pagán R., Condón S. (2012). UV-C inactivation of *Cronobacter sakazakii*. Foodborne Pathog. Dis..

[60] Gayán E., Serrano M.J., Raso J., Álvarez I., Condón S. (2012). Inactivation of *Salmonella enterica* by UV-C light alone and in combination with mild temperatures. Appl. Environ. Microbiol..

[61] Gouma M., Gayán E., Raso J., Condón S., Álvarez I. (2015). Inactivation of spoilage yeasts in apple juice by UV-C light and in combination with mild heat. Innov. Food Sci. Emerg. Technol..

[62] Gabriel A.A., David M.M.C., Elpa M.S.C., Michelena J.C.D. (2020). Decontamination of dried whole black peppercorns using ultraviolet-c irradiation. Food Microbiol..

[63] Islam M.S., Patras A., Pokharel B., Vergne M.J., Sasges M., Begum A., Rakariyatham K., Pan C., Xiao H. (2016). Effect of UV irradiation on the nutritional quality and cytotoxicity of apple juice. J. Agric. Food Chem..

[64] Guneser O., Karagul Yuceer Y. (2012). Effect of ultraviolet light on water- and fat-soluble vitamins in cow and goat milk. J. Dairy Sci..

[65] Molina B., Sáez M.I., Martínez T.F., Guil-Guerrero J.L., Suárez M.D. (2014). Effect of ultraviolet light treatment on microbial contamination, some textural and organoleptic parameters of cultured sea bass fillets (*Dicentrarchus labrax*). Innov. Food Sci. Emerg. Technol..

[66] Matak K.E., Sumner S.S., Duncan S.E., Hovingh E., Worobo R.W., Hackney C.R., Pierson M.D. (2007). Effects of ultraviolet irradiation on chemical and sensory properties of goat milk. J. Dairy Sci..

[67] Ghate V., Kumar A., Zhou W., Yuk H.G. (2016). Irradiance and temperature influence the bactericidal effect of 460-nanometer light-emitting diodes on *Salmonella* in orange juice. J. Food Prot..

[68] Kim M.J., Bang W.S., Yuk H.G. (2017). 405 ± 5 nm light emitting diode illumination causes photodynamic inactivation of *Salmonella* spp. on fresh-cut papaya without deterioration. Food Microbiol..

[69] Kim M.J., Tang C.H., Bang W.S., Yuk H.G. (2017). Antibacterial effect of 405 ± 5 nm light emitting diode illumination against *Escherichia coli* O157:H7, *Listeria monocytogenes*, and *Salmonella* on the surface of fresh-cut mango and its influence on fruit quality. Int. J. Food Microbiol..

